# A Hyperfluorinated Hydrophilic Molecule for Aqueous ^19^F MRI Contrast Media

**DOI:** 10.1155/2018/1693513

**Published:** 2018-11-12

**Authors:** Eric A. Tanifum, Laxman Devkota, Conelius Ngwa, Andrew A. Badachhape, Ketan B. Ghaghada, Jonathan Romero, Robia G. Pautler, Ananth V. Annapragada

**Affiliations:** ^1^Department of Pediatric Radiology, Texas Children's Hospital, Houston, TX 77030, USA; ^2^Department of Radiology, Baylor College of Medicine, Houston, TX 77030, USA; ^3^Department of Molecular Physiology and Biophysics, Baylor College of Medicine, TX 77030, USA

## Abstract

Fluorine-19 (^19^F) magnetic resonance imaging (MRI) has the potential for a wide range of *in vivo* applications but is limited by lack of flexibility in exogenous probe formulation. Most ^19^F MRI probes are composed of perfluorocarbons (PFCs) or perfluoropolyethers (PFPEs) with intrinsic properties which limit formulation options. Hydrophilic organofluorine molecules can provide more flexibility in formulation options. We report herein a hyperfluorinated hydrophilic organoflourine, **ET1084**, with ∼24 wt. % ^19^F content. It dissolves in water and aqueous buffers to give solutions with ≥8 M ^19^F. ^19^F MRI phantom studies at 9.4T employing a 10-minute multislice multiecho (MSME) scan sequence show a linear increase in signal-to-noise ratio (SNR) with increasing concentrations of the molecule and a detection limit of 5 mM. Preliminary cytotoxicity and genotoxicity assessments suggest it is safe at concentrations of up to 20 mM.

## 1. Introduction

MRI is currently the most powerful technique devoid of ionizing radiation for noninvasive clinical interrogation of the state of disease in soft tissue. Following the report of the first proton (^1^H) MRI in 1973 [1], the technique quickly underwent several technological advances. Today, high resolution 3D anatomical images of all soft tissue types [2] can be obtained routinely in clinics across the globe. Obtaining medical information at the cellular and molecular levels by ^1^H MRI often requires the use of contrast agents, and a variety of these are currently in use [3]. ^1^H MRI contrast agents (CAs) generate contrast *in vivo* by altering the relaxivity of ^1^H spins in surrounding water molecules but suffer from low SNR due to high background signal from water in soft tissue [4].

The first ^19^F MRI images were reported in 1977 [5], but the platform received little attention as a clinical imaging technique until 2005 when Ahrens et al. demonstrated its potential for *in vivo* cell tracking [6]. Since then, several exogenous PFC and PFPE probes have been used successfully to track different cell types *in vivo* by ^19^F MRI. These include dendritic cells (DCs) in humans [7], T cell studies to track inflammatory events in a rodent model of type 1 diabetes [8], endogenous monocytes and macrophages in inflammatory lesions [9], macrophage distribution and density in mammary tumors and lung metastases [10], as well as lung imaging [11]. Other applications such as molecular imaging of thrombus and angiogenesis [12] have also been assessed.

^19^F MRI contrast agents are superior to ^1^H MRI because there is no endogenous MR detectable ^19^F in soft tissue. There is therefore negligible tissue background signal, resulting in images with superior SNR. Recent advances in ^19^F MRI technology including improvements in radiofrequency (RF) coil design, the development of dual ^19^F/^1^H imaging, as well as more advanced scan protocols [13] have greatly reduced scan times and improved image processing. Instruments with the ability to simultaneously capture complementary high resolution anatomical ^1^H MR images alongside ^19^F MRI hot-spots in one imaging session are also currently available [14]. However, the advances in instrumentation, scan protocols, and image analysis have not been matched by similar developments in biocompatible exogenous ^19^F probes with flexible/diverse *in vivo* applications [15]. Apart from PFCs and PFPEs, several other fluorinated CAs including dendrimers, fluorinated amphiphiles, and hyperfluorinated molecules (such as **PERFECTA**) have been reported. These were examined in a recent review by Tirotta et al. [16].

To date, PFCs and PFPEs constitute the most common ingredient in exogenous ^19^F MRI probes. However, their high hydrophobicity limits formulation flexibility and wide *in vivo* applicability. More recent research efforts in fluorinated CAs are gradually shifting towards hydrophilic molecules due to perceived flexibility in formulation and applicability. Several fluorinated hydrophilic polymers containing up to 20 % fluorinated monomer units while maintaining water solubility have been reported [17–20]. However, these commonly have a ^19^F content of <5 wt. %. Zhang et al. recently reported a hydrophilic PFPE-based polymer with a ^19^F content of ∼30 wt. % (albeit with magnetic resonances spread across ∼65 ppm units) [21]. In a recent report, we demonstrated that small hydrophilic nonionic organofluorine molecules have the potential for facile formulation into a myriad of ^19^F MRI probes with unique ^19^F MR signatures [22]. We present herein the synthesis and characterization of a new hyperfluorinated nonionic organofluorine molecule, **ET1084**, with ^19^F content of ∼24 wt. %. This compound dissolves in water and aqueous buffers, yielding solutions of ≥8 M ^19^F atoms which generate ^19^F MR images with no chemical shift artifacts. Preliminary evaluation of cytotoxicity in RAW264.7 (a monocyte cell line) and ImKC (a Kupffer cell line) cells and genotoxicity in *E. coli* suggests that it is nontoxic at concentrations of up to 20 mM.

## 2. Materials and Methods

### 2.1. Chemical Synthesis

#### 2.1.1. General Procedures

Procedures similar to our previously reported [23] general chemical synthesis procedures were employed. 2,2,3,3-Tetrafluoro-1,4-butanediol was purchased from Exfluor Research Corp., Round Rock, TX, USA. All other reagents were obtained from Sigma-Aldrich and used without further purification. Proton nuclear magnetic resonance (^1^H NMR) spectra were recorded at 600 MHz on a Bruker 600 NMR spectrometer or at 300 MHz on a Bruker 300 NMR spectrometer. Carbon nuclear magnetic resonance (^13^C NMR) spectra were recorded at 151 MHz on a Bruker 600 NMR spectrometer or at 75 MHz on a Bruker 300 NMR spectrometer. Fluorine nuclear magnetic resonance (^19^F NMR) spectra were recorded at 282 MHz on a Bruker 300 NMR spectrometer. Chemical shifts are reported in parts per million (ppm) from an internal standard of acetone (2.05 ppm), chloroform (7.26 ppm), or water (4.79 ppm) for ^1^H NMR and from an internal standard of either residual acetone (206.26 ppm), chloroform (77.00 ppm), or dimethyl sulfoxide (39.52 ppm) for ^13^C NMR. NMR peak multiplicities are denoted as follows: s (singlet), d (doublet), t (triplet), q (quartet), p (pentet), bs (broad singlet), dd (doublet of doublet), tt (triplet of triplet), ddd (doublet of doublet of doublet), and m (multiplet). Coupling constants (*J*) are given in hertz (Hz). High resolution mass spectra (HRMS) were obtained from the Mass Spectrometry Unit of the Bioscience Research Collaborative at Rice University, Houston, Texas. Thin layer chromatography (TLC) was performed on silica gel 60 F254 plates from EMD Chemical Inc., and components were visualized by ultraviolet light (254 nm) and/or phosphomolybdic acid, 20 wt.% solution in ethanol. SiliFlash silica gel (230–400 mesh) was used for all column chromatography.

#### 2.1.2. Compound 1: Bis(2,2-dimethyl-1,3-dioxolan-4-yl)methanol

To a mixture of xylitol (50.0 g, 329 mmol), dimethoxy acetone (60.0 mL), and methanol (100 mL) in acetone (2000 mL), *p*-toluenesulfonic acid monohydrate (5.65 g, 32.8 mmol) was added followed by vigorous stirring. All solids were dissolved after vigorous stirring for approximately 3 h, and the reaction mixture was stirred at room temperature overnight. K_2_CO_3_ (4.54 g, 32.8 mmol) was added and stirred for 30 mins. Undissolved solids were filtered off, and the ensuing filtrate was concentrated *in vacuo.* The resultant colorless oil was chromatographed on silica gel eluted with 40% ethyl acetate/pentane to yield secondary alcohol **1** (64.8 g, 279 mmol, 85%) as a colorless oil. ^1^H NMR (600 MHz, CDCl_3_): *δ* ppm 4.18−4.15 (m, 1H), 4.02 (t, *J* = 6.9 Hz, 2H), 3.95−3.93 (m, 1H), 3.83 (t, *J* = 7.7 Hz, 1H), 3.77 (dd, *J* = 12.0, 1.7 Hz, 1H), 3.61 (dd, *J* = 12.0, 4.3 Hz, 1H), 1.40 (s, 9H), 1.35 (s, 3H). ^13^C NMR (151 MHz, CDCl_3_): *δ* ppm 109.7, 109.6, 77.7, 75.1, 65.9, 65.6, 62.1, 27.1, 26.9, 26.1, 25.4. HRMS *clcd* for C_11_H_20_O_5_^+^ *m*/*z* [M + Na]^+^ 255.1203, found 255.1207.

#### 2.1.3. Compound 2: Bis(2,2-dimethyl-1,3-dioxolan-4-yl)methyl Methane Sulfonate

To a solution of the alcohol **1** (50.0 g, 215 mmol) in CH_2_Cl_2_ (1000 mL) under N_2_ atmosphere, trimethylamine (90.0 mL, 646 mmol) was added and the resultant solution was cooled to 0°C for 15 mins. Methanesulfonyl chloride (21.7 mL, 280 mmol) was added, and the mixture was warmed to ambient temperature with stirring for 1 h after which the reaction was shown to be complete by TLC. The mixture was poured into saturated NH_4_Cl solution (1000 mL) with a separatory funnel. The organic phase was separated, and the aqueous phase was re-extracted with CH_2_Cl_2_. The combined organic extracts were rinsed with brine, dried over Na_2_SO_4_, and concentrated by rotary evaporation to obtain a brown paste which precipitated upon addition of diethyl ether. The precipitate was filtered to give mesylate **2** (41.9 g, 176 mmol, 82%) as a pale yellow crystalline solid. ^1^H NMR (600 MHz, CDCl_3_): *δ* 4.43 (dd, *J* = 11.2, 3.0 Hz, 1H), 4.30 (dd, *J* = 11.2, 5.3 Hz, 1H), 4.28-4.24 (m, 2H), 4.10 (t, *J* = 7.7 Hz, 1H), 4.01 (dd, *J* = 8.1, 3.9 Hz, 1H), 3.93 (t, *J* = 7.6 Hz, 1H), 3.10 (s, 3H), 1.46 (s, 3H), 1.45 (s, 6H), 1.39 (s, 3H). ^13^C NMR (151 MHz, CDCl_3_): *δ* ppm 110.5, 109.9, 76.5, 75.0, 74.2, 68.8, 65.4, 37.7, 27.0, 26.9, 26.1, 25.2. HRMS *clcd* for C_12_H_22_O_7_S^+^ *m*/*z* [M + H]^+^ 311.1159, found 311.1151.

#### 2.1.4. Compound 3: 4-[Bis(2,2-dimethyl-1,3-dioxolan-4-yl)methoxy]-2,2,3,3-tetrafluorobutan-1-ol

To a suspension of sodium hydride powder (12.3 g, 308 mmol, 60% in mineral oil) in dry diglyme (1000 mL) at 0°C, 2,2,3,3-tetrafluoro-1,4-butanediol (25.0 g, 154 mmol) was added slowly. The ensuing mixture was stirred at 0°C for 1 h under N_2_ atmosphere. To the resultant alkoxide, mesylate **2** (19.2 g, 61.7 mmol) was added and the reaction mixture was heated at 90°C for 8 h. The resultant dark solution was carefully poured into ice water solution, and all volatile materials were stripped off by rotary evaporation under reduced pressure. The resulting brown solid was purified by flash column chromatography eluted with 10–55% ethyl acetate/hexanes gradient to yield alcohol **3** (13.0 g, 34.6 mmol, 56%) as a colorless oil. ^1^H NMR (600 MHz, CDCl_3_): *δ* ppm 4.21 (q, *J* = 5.7 Hz, 1H), 4.14 (dt, *J* = 8.3, 4.2 Hz, 1H), 4.07 (q, *J* = 7.3 Hz, 1H), 4.01 (qd, *J* = 13.5, 6.8 Hz, 4H), 3.94 (dd, *J* = 8.1, 4.2 Hz, 1H), 3.90 (t, *J* = 7.7 Hz, 1H), 3.82 (dd, *J* = 10.4, 3.5 Hz, 1H), 3.73 (dd, *J* = 10.4, 5.4 Hz, 1H), 3.02 (t, *J* = 7.6 Hz, 1H), 1.45 (s, 6H), 1.44 (s, 3H), 1.39 (s, 3H). ^19^F NMR (282 MHz, CDCl_3_) *δ* ppm 122.0, 124.2. (^13^C NMR (151 MHz, CDCl_3_): *δ* ppm 110.1, 109.8, 77.2, 76.0, 74.8, 72.6, 68.3 (t, *J* = 28.19), 65.5, 60.5 (t, *J* = 28.21), 26.9, 26.1, 25.3. HRMS *clcd* for C_15_H_24_F_4_O_6_^+^ *m*/*z* [M + H]^+^ 377.1582, found 377.1585.

#### 2.1.5. Compound 4: 1,2,3,4,5,6-Hexakis{4-[bis(2,2-dimethyl-1,3-dioxolan-4-yl)methoxy]-2,2,3,3-tetrafluorobutoxy}methyl)benzene

To a suspension of sodium hydride powder (2.17 g, 55.2 mmol, 60% in mineral oil) in dry THF (200 mL) at 0°C, alcohol **3** (9.50 g, 25.2 mmol) was added slowly and stirred at 0°C for 1 h under N_2_ atmosphere. Hexakis(bromomethylbenzene) (2.47 g, 3.88 mmol) was added and stirred for 12 h at room temperature. The reaction mixture was carefully poured into ice water solution, and the resultant solution evaporated under reduced pressure to obtain a brown solid residue. This was purified using flash column chromatography (15–60% ethyl acetate/hexanes) to yield compound **4** (8.35 g, 3.47 mmol, 89%) as a pale yellow syrup. ^1^H NMR (600 MHz, CDCl_3_): *δ* 4.85 (s, 12H), 4.19 (q, *J* = 5.8 Hz, 6H), 4.10 (dt, *J* = 8.1, 4.2 Hz, 6H), 4.02 (m, 30H), 3.95 (dd, *J* = 7.9, 4.6 Hz, 6H), 3.86 (t, *J* = 7.8 Hz, 6H), 3.78-3.71 (m, 12H), 1.44 (s, 36H), 1.43 (s, 18H), 1.39 (s, 18H). ^19^F NMR (282 MHz, CDCl_3_): *δ* ppm −121.1, −121.3. ^13^C NMR (151 MHz, CDCl_3_): *δ* ppm 137.7, 109.9, 109.7, 77.7, 76.4, 75.2, 72.8, 68.2, 68.0, 67.8, 65.6, 26.9, 26.2, 25.4. HRMS *clcd* for C_102_H_150_F_24_O_36_^+^ *m*/*z* [M + Na]^+^ 2429.9416, found 2429.9470.

#### 2.1.6. Compound

**ET1084**. Compound **4** (6.80 g, 2.80 mmol) was dissolved in a THF/6M HCl mixture (1 : 1, 100 mL) and refluxed at 80°C for 3 h. The solvents were evaporated, and the mixture was diluted with water (20.0 mL) and neutralized by adding NaOH solution (2M) dropwise. The resulting mixture was freeze-dried, and inorganic salts were removed by dissolving in dry ethanol and filtering off undissolved solids. The ethanol from the filtrate was stripped by rotary evaporation, and the ensuing clear paste was diluted with water and freeze-dried to obtain **ET1084** (4.75 g, 2.47 mmol, 88%) as a white solid. ^1^H NMR (600 MHz, MeOD): *δ* 4.94 (s, 12H), 4.11 (t, *J* = 14.9 Hz, 12H), 4.00 (t, *J* = 14.3 Hz, 12H), 3.90 (d, *J* = 4.2 Hz, 6H), 3.75 (dd, *J* = 9.4, 4.3 Hz, 12H), 3.68 (dt, *J* = 10.9, 5.6 Hz, 12H), 3.63 (t, *J* = 4.8 Hz, 12H). ^13^C NMR (151 MHz; MeOD): 137.7, 73.9, 42.4, 70.9, 70.6, 67.8 (t, 25 Hz) 67.2 (t, 24.9 Hz), 62.9, 61. 4. ^19^F NMR (282 MHz; MeOD): *δ* −122.7, −123.0. HRMS *clcd* for C_66_H_102_F_24_O_36_^+^ *m*/*z* [M + Na]^+^ 1949.5665, found, 1949.5660.

### 2.2. MRI Acquisition and Data Processing

All MRI scans were performed on a 9.4T Bruker small animal MR scanner equipped with a ^1^H/^19^F dual-tunable volume RF coil (35 mm inner diameter, 50 mm length; Rapid Biomed, Würzburg, Germany), located in the Small Animal Imaging Facility (SAIF) at Texas Children's Hospital. ^19^F images of phantoms were acquired with an MSME scan protocol (excitation bandwidth = 2000 Hz, TR = 2000 ms, TE = 8.95 ms, and scan time = 10 min 40 s). DICOMs obtained from scans were processed using the OsiriX v.5.8.5 software (Pixmeo SARL, Bernex, Switzerland).

### 2.3. Relaxation times

Relaxation times *T*_1_ and *T*_2_ were estimated using similar scan sequences and parameters as previously reported [22]: for *T*_1_ relaxation times, a saturation recovery (RAREVTR) sequence with the following parameters (FOV = 5 ∗ 5 cm^2^; matrix = 32 ∗ 32; slices = 14; ST = 0.7 mm; TE = 11 ms; TR = 10000, 5000, 2500, 1500, 800, 400, 200, 100 ms; rare factor = 2; BW = 15 kHz; NA = 50; dummy scans (DSs) = 0); for *T*_2_ relaxation times, a multislice multiecho (MSME) sequence with the following parameters (FOV = 5 ∗ 5 cm^2^; matrix = 32 ∗ 32; slices = 1; slice thickness = 10 mm; TE = 11 ms; TR = 5000 ms; number of echos = 40; BW = 15 kHz; NA = 100; DS = 0). Image sequence analysis in ParaVision 5.1 software was used to convert the raw data to numerical values.

### 2.4. Toxicity Assays

#### 2.4.1. Materials

All materials were purchased from vendors, including DPBS, DMEM (containing 4.5 g/L glucose, L-glutamine, and sodium pyruvate), FBS, and 0.25% trypsin containing 2.21 mM EDTA (Corning Cellgro, Manassas, VA, USA); LPS from *E. coli* 0111: B4 and BMH-21 (Sigma Aldrich, St. Louis, MO, USA); and penicillin (10,000 U/mL)/streptomycin (10,000 *µ*g/mL) (Lonza, Walkersville, MD, USA). Mouse RAW264.7 macrophages were obtained from ATCC, ImKCs (immortalized Kupffer cells) were obtained from EMD Millipore Corporation (Temecula, CA, USA), and *E. coli* SOS-Chromotest kit was obtained from EBPI (Mississauga, Ontario, Canada).

#### 2.4.2. Cell Culture

RAW264.7 cells were cultured in DMEM containing 4.5 g/L glucose, L-glutamine, and sodium pyruvate and supplemented with 10% heat-activated FBS and 1% P/S. ImKCs were cultured in a RMPI-1640 medium containing L-glutamine and supplemented with 10% heat-inactivated FBS and 1% P/S. *E. coli* was cultured in a growth medium supplied with the SOS-Chromotest kit. All cells were incubated in a humidified atmosphere with 5% CO_2_ at 37°C.

#### 2.4.3. Cytotoxicity: MTS/PMS Assay for Determination of LC50 and Cell Viability

RAW264.7 or ImKCs (0.01 × 10^6^ cells/well) were plated in then treated at different concentrations of **ET1084**, BMH-21 (positive control), or left untreated (negative control) for 24 h. Dehydrogenase activity in the cultured cells was assayed using CellTiter 96R Aqueous Non-Radioactive Cell Proliferation assay kit obtained from Promega (Madison, WI, USA), according to the manufacturer's protocol. Briefly, cells were washed with PBS and then dosed with combined MTS/PMS solution in a fresh culture medium. Following incubation of the dosed cells for 4 hours at 37°C, the absorbance was measured immediately at *λ* = 450 nm using a multimode microplate reader (Filter Max F5, Molecular Devices).

#### 2.4.4. Genotoxicity

The genotoxic potential of **ET1084** was determined using the SOS-Chromotest version 6.5 obtained from EBPI (Mississauga, Ontario, Canada) with modification. Briefly, *E. coli* was hydrated in a growth medium and incubated for 15 h in a humidified atmosphere with 5% CO_2_ at 37°C. The absorbance (at 600 nm) of the turbid overnight bacteria suspension was adjusted to 0.055 using a fresh growth medium. The bacterial suspension (0.055 OD, 100 mL) was homogenized with or without **ET1084** at final concentrations of 0, 10, 15, 20, and 30 mM or 4-NQO (positive control) content in a 96-welled plate. Following 2 h incubation under conditions mentioned earlier, blue chromogen substrate (p-nitrophenyl phosphate in blue chromogen solution, 100 *µ*L) solution was added to the inoculated medium and further incubated for 1.5 h. The experiment was quenched by addition of stop solution (50 *µ*L), and genotoxicity (*β*-galactosidase activity) was measured at 595 nm; meanwhile, viability (alkaline phosphatase activity) was measured at 405 nm using a multimode microplate reader (Filter Max F5, Molecular Devices). The induction factor (IF) which is a correlation of the *β*-galactosidase and alkaline phosphatase activities was used to define the degree of genotoxicity [24, 25].

#### 2.4.5. Statistical Analysis

Experiments were performed in triplicate, and the mean was calculated with standard deviation. Dose-response data for LC50 determination were analyzed by the probit method using Finney's table [26]. The LC50 values for RAW264.7 and ImKCs were determined by applying regression equation analysis to the probit-transformed data of mortality using Excel spreadsheet [27].

## 3. Results and Discussion

**Scheme 1:**
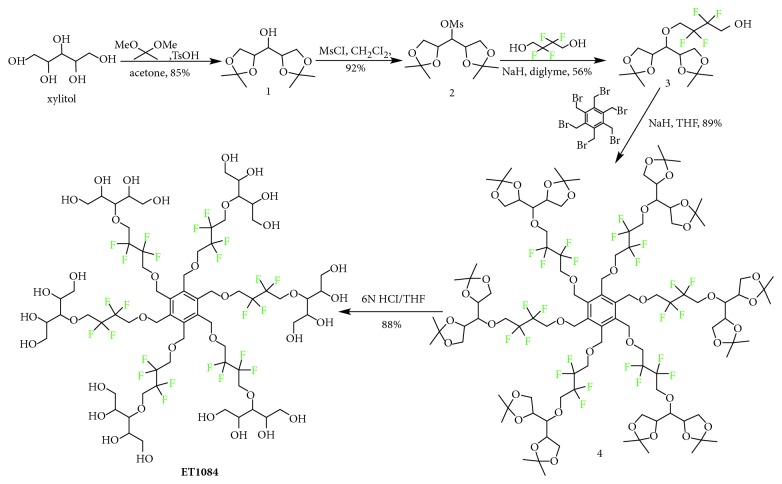
Synthetic route to **ET1084**.

**ET1084** was designed to have a tertiary ring conformation with a hydrophilic surface composed of highly soluble and biocompatible xylitol over a hydrophobic core bearing ^19^F atoms with close-to-identical magnetic resonance frequencies. The close-to-identical magnetic resonances are a requisite to ^19^F MR images devoid of chemical shift artifacts [14]. As shown in Scheme 1, the compound was readily accessed from xylitol in four high-yielding synthetic steps. First, the terminal vicinal alcohols of xylitol were capped with acetone using standard acetonide protection conditions to obtain alcohol **1**. The hydroxyl group of **1** was activated to obtain mesylate **2**, which was heated in the presence of excess preformed 2,2,3,3-tetrafluoro-1,4-butanedialkoxide to obtain compound **3**. The alkoxide of compound 3 reacted with Hexakis(bromomethylbenzene) to obtain compound **4**, which was deprotected under acid conditions to obtain **ET1084**. The structures of all intermediates and the final product were confirmed by ^1^H NMR, ^19^F NMR (where applicable), and ^13^C NMR as well as HRMS.

**Figure 1:**
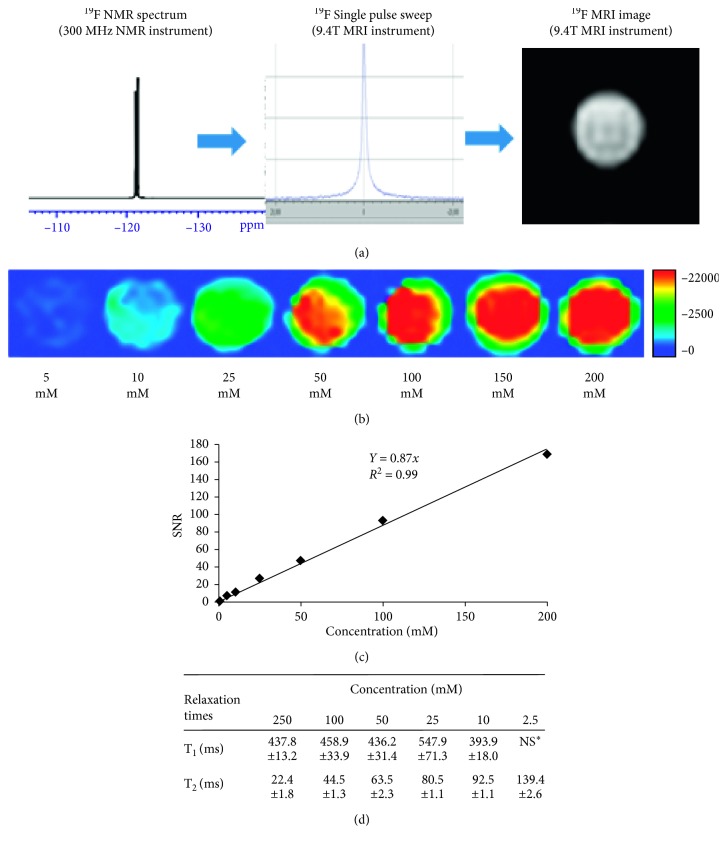
Magnetic resonance characteristics of **ET1084**. (a) Proton decoupled ^19^F NMR spectrum from a 300 MHz instrument shows two peaks separated by 0.3 ppm and a single peak from a single pulse frequency sweep in a 9.4T small animal imaging instrument which generates a sharp ^19^F MRI image devoid of chemical shift artifacts; (b) dilution studies show that the compound is visible at concentrations of 5 mM from a 10-minute MSME scan at 9.4T, (c) plot of SNR against concentration shows linearity (*R* ^2^ = 0.99); (d) *T*_1_ and *T*_2_ relaxation times estimated by ^19^F MRI at 9.4T (details on scan parameters in SI). Data reported as mean ± SD.

**ET1084** dissolves readily in water and aqueous buffers including phosphate-buffered saline (PBS), histidine/saline, and acetate buffers to give clear solutions of ≥8 M ^19^F at room temperature. The ^19^F NMR spectrum (Figure 1(a)) shows two peaks at −121.4 ppm (separated by 0.3 ppm units). A single pulse frequency sweep from a 9.4T small animal imaging instrument shows a single peak which leads to ^19^F MRI images with no chemical shift artifacts. Phantom dilution studies using 1T and 9.4T instruments (see Supporting Materials S2) suggested that a concentration range between 0 and 200 mM was optimal for the characterization of the molecule at 9.4T. ^19^F MRI scans performed on solutions of the molecule at concentrations of 1, 5, 10, 25, 50, 100, and 200 mM (Figure 1(b)), employing a 10-minute multislice multiecho (MSME) scan protocol (excitation bandwidth = 2000 Hz, TR = 2000 ms, and TE = 8.95 ms), showed a clear signal at concentrations as low as 5 mM with an observed SNR of 8.4 at this concentration. A plot of SNR against concentration (Figure 1(c)) showed a linear relationship (*r* ^2^ = 0.99) at concentrations up to 200 mM. Beyond this concentration (see Supporting Materials S2), the SNR starts dropping. Analyses of the spin-lattice relaxation time (*T*_1_) and the spin-spin relaxation time (*T*_2_) at different concentrations of the molecule (Figure 1(d)) suggest a *T*_1_ of ∼450 ms and T_2_s, which show significant shortening with increasing concentration. This drop in SNR at concentrations >200 mM is rationalized by the spin-echo pulse sequence employed in acquiring the images. In ^19^F MRI, using a spin-echo scan protocol, the signal intensity (I) can be related to the number of ^19^F nuclei (N), the relaxation times (*T*_1_ and *T*_2_), and the scan parameters TR (repetition time) and TE (echo time) by [28]

(1) $$\begin{array}{c} I=N\left(F\right)\left[1-2e^{-\left(TR-TE/2\right)/\left(2T_{1}\right)}+e^{-TR/T_{1}}\right]e^{-TE/T_{2}}. \end{array}$$

It should be noted that in an aqueous medium where the concentration of water is ∼55 M compared to millimolar concentrations of the molecule, the spin-lattice relaxation time, *T*_1_, will remain fairly constant within the millimolar range for the ^19^F nuclei. However, the spin-spin relaxation time, *T*_2_, determined by the strength of dipolar interaction between neighboring ^19^F nuclei and proton nuclei, experiences more significant changes with every increase in concentration of ^19^F species. An increase in dipolar interactions between ^19^F nuclei with increasing concentration causes faster relaxation of the nuclei. This in turn translates to the observed shortening in T_2_s. Based on Equation (1), this should lead to a decrease in signal intensity. However, the signal intensity is also directly proportional to the number of ^19^F nuclei, so an increase in concentration should result in a corresponding increase in signal intensity. The overall effect on the signal intensity is therefore a trade-off between *T*_2_ and effective concentration of ^19^F nuclei. The observed increase in SNR with increasing concentration up to 200 mM and drop thereof suggest that the effect on the signal intensity is dominated by the effective concentration of ^19^F nuclei within this range and the *T*_2_ effect dominates thereafter.

**Figure 2:**
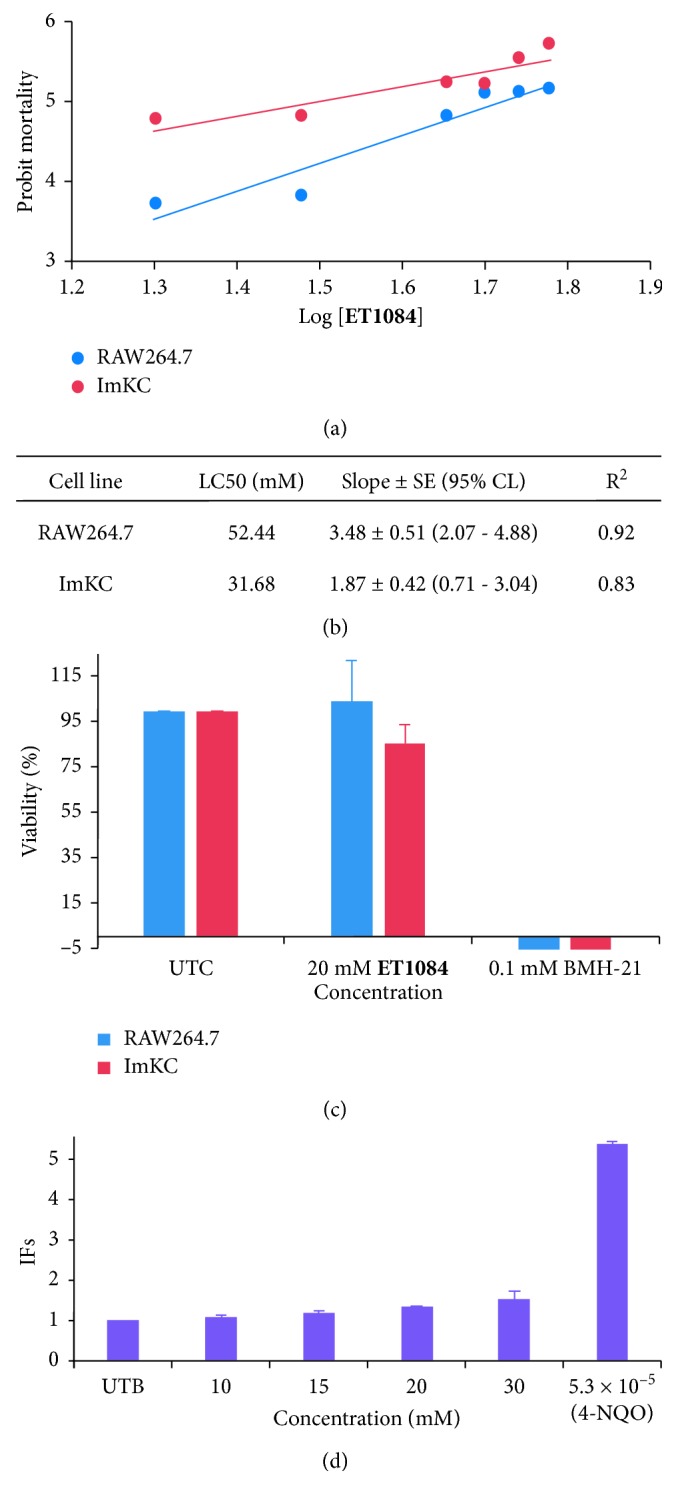
Preliminary toxicity evaluation of **ET1084**. (a) Probit analysis to determine LC50 values of **ET1084** against RAW264.7 and ImKCs; (b) LC50 values, slope, the corresponding 95% confidence limit (CL), and goodness of fit (*R* ^2^); (c) cell viability of both cell lines treated with 20 mM **ET1084** compared to untreated cells and 0.1 mM BMH-21 (positive control); (d) IFs plots following treatment of *E. coli* with different concentrations of **ET1084** and 4-NQO (positive control). IFs < 1.5 indicate no genotoxicity.

For *in vivo* applications, high concentrations of **ET1084** are required at the target to allow conspicuity and this is achievable by nanoparticle formulation. Its high aqueous solubility makes liposome formulation an attractive option. Liposomes are cleared through the monocyte-phagocyte system (MPS). As a result, leucocytes and resident immune cells in the liver and spleen are more likely to encounter high concentrations of the compound. We therefore chose a monocyte cell line and a Kupffer cell line to preliminarily assess the toxicity of the compound. RAW264.7 (monocyte cell line) and ImKCs (Kupffer cell line) were exposed to different concentrations of **ET1084** over 24 hours. Regression graphs of probit mortality of both cell types plotted against log values of increasing concentrations of the compound (Figure 22(a)) suggest that the LC50 of the compound is 52.44 mM for the RAW264.7 cells and 31.68 mM for the ImKCs. In addition, the data suggest that the compound is innocuous to the RAW264.7 at concentrations up to 20 mM and cell viability of over 80 % was observed for the ImKCs at the same concentration (Figure 2(c)).

The potential of **ET1084** to react with genetic material was also assessed using the SOS-Chromotest kit, which allows for the detection of DNA-damaging agents in *Escherichia coli (E. coli)*.  Figure 2(d) shows *β*-galactosidase induction factors (IFs) following incubation of *E. coli* with various concentrations of **ET1084** or 4-nitroquinoline-1-oxide (4-NQO) as a positive control. IFs < 1.50 were observed for concentrations of **ET1084** up to 20 mM, and 1.5 was recorded at a concentration of 30 mM. For this assay, a test sample with IF < 1.5 is considered nongenotoxic, 1.5 ≤ IF ≤ 2 is marginally genotoxic, and IF >2 is genotoxic [29, 30].

## 4. Conclusion

In summary, we have synthesized and characterized a novel hydrophilic hyperfluorinated organofluorine molecule amenable to aqueous formulations of exogenous probes for *in vivo* ^19^F MRI applications. Preliminary toxicity assessment suggests that the compound is safe at concentrations up to 20 mM, well above the detectable threshold of 4 mM. This suggests that safe formulations of this compound are accessible for *in vivo* applications. The high aqueous solubility of the compound (≥8 M ^19^F solutions) suggests that it can be formulated into liposomes (a biocompatible and versatile nanoparticle platform) with a payload capacity of 24 million ^19^F atoms per particle for a formulation with a mean particle diameter of 150 nm. The preparation of both targeted and nontargeted liposome formulations of this compound for *in vivo* assessment is ongoing and will be reported in the future.

## Data Availability

The data used to support the findings of this study are available from the corresponding author upon request.

## Supplementary Materials

Supplementary Materials S1: ^1^H, ^13^C, and ^19^F NMR spectra of all compounds. S2: determination of the optimal concentration range to evaluate the ^19^F MRI properties of **ET1084**.
