# Supplementary material for: A Hyperfluorinated Hydrophilic Molecule for Aqueous 19F MRI Contrast Media
Source: Contrast Media Mol Imaging. 2018 Nov 12;2018:1693513. doi: 10.1155/2018/1693513 (PMC6260405; doi:10.1155/2018/1693513)
Supplement: Supplementary Materials — S1: 1H, 13C, and 19F NMR spectra of all compounds. S2: determination of the optimal concentration range to evaluate the 19F MRI properties of ET1084. [file 1693513.f1.pdf]

Supporting Information

A hyperfluorinated hydydrophilic molecule for aqueous ^{19}F MRI contrast media

Eric A. Tanifum[†,‡,*], Laxman Devkota[‡], Conelius Ngwa[‡], Andrew A. Badachhape[‡], Ketan B. Ghaghada[†,‡], Jonathan Romero[§], Robia A. Pautler[‡,§], Ananth V. Annapragada[†,‡].

[†]Department of Pediatric Radiology, Texas Children's Hospital, Houston, TX 77030, USA.
[‡]Department of Radiology, Baylor College of Medicine, Houston, TX 77030, USA.
[§]Department of Molecular Physiology and Biophysics, Baylor College of Medicine, TX 77030, USA.

*To whom correspondence should be addressed: E-mail: eatanifu@texaschildrens.org.
ORCID: 0000-00018803-5643

Contents

^{1}H, ^{13}C and ^{19}F NMR Spectra of Compounds………………………………………………..….**S1**

Determination of optimal concentration range to evaluate the ^{19}F MRI properties of **ET1084**...**S2**

S1. ^{1}H, ^{13}C and ^{19}F NMR Spectra of Compounds

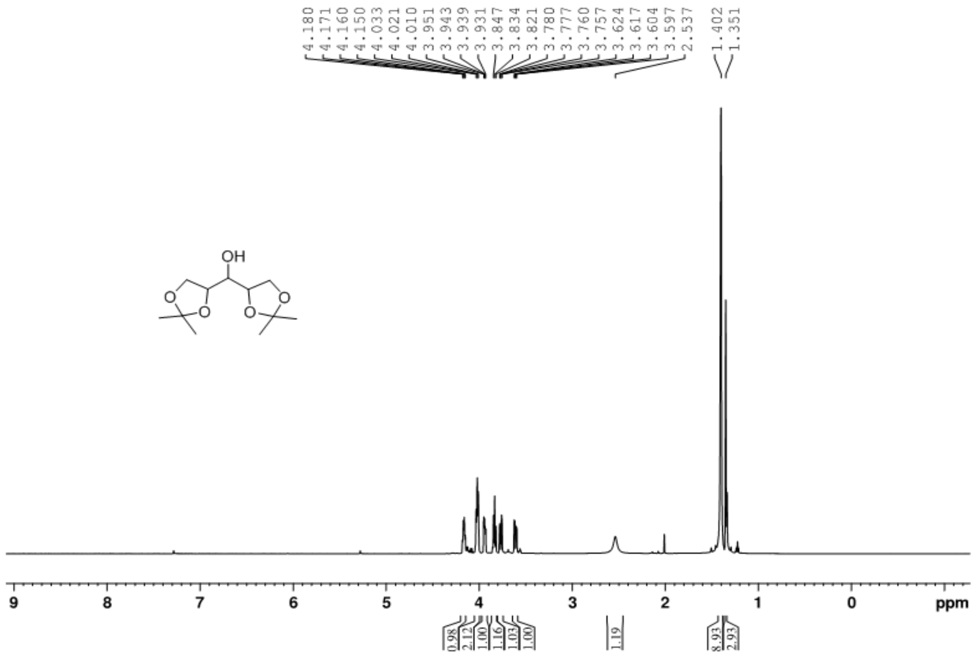

^{1}H NMR of Compound **1**

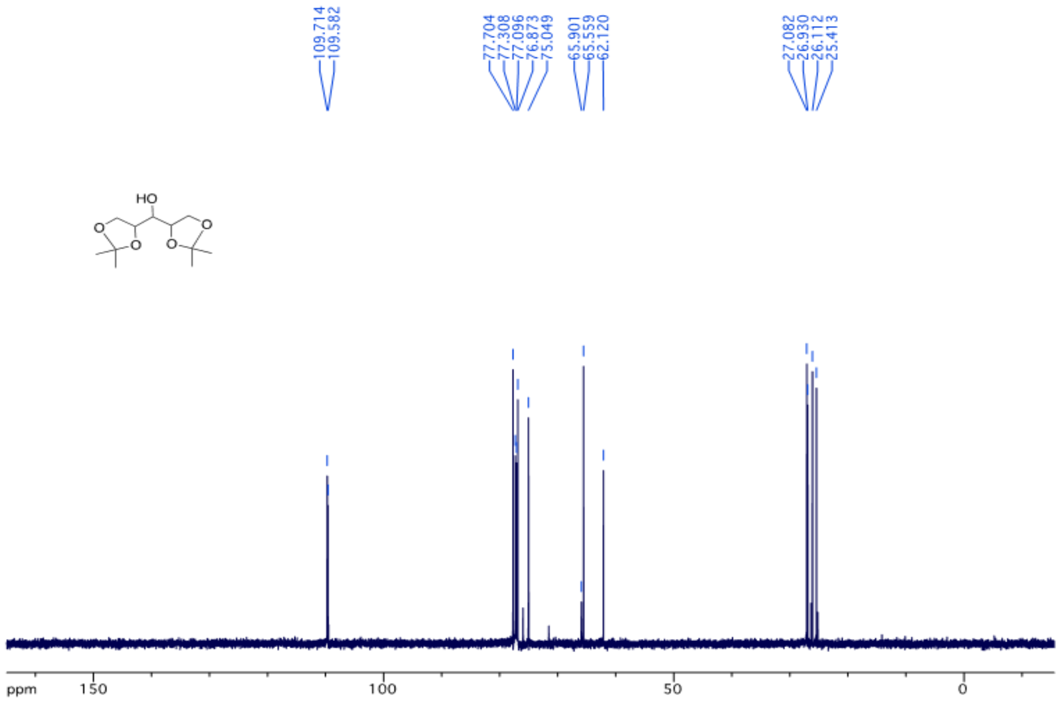

^{13}C NMR of Compound **1**

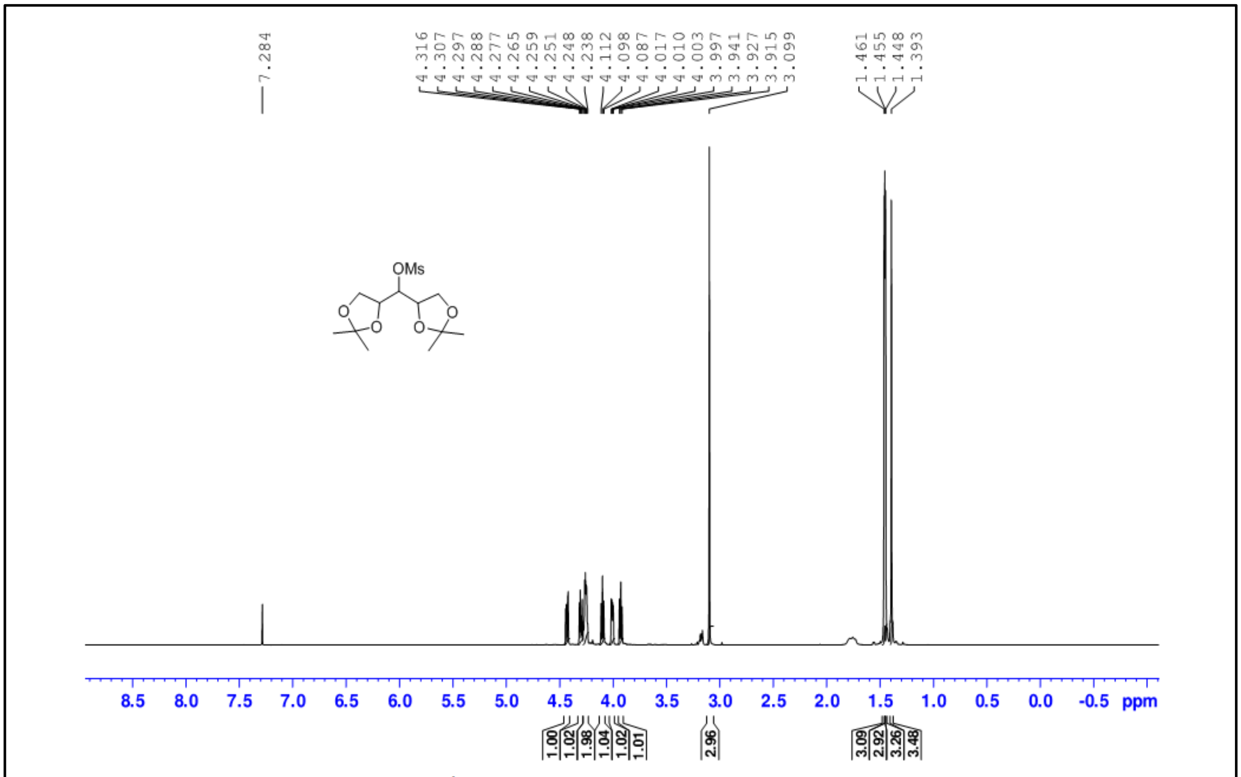

^{1}H NMR of Compound **2**

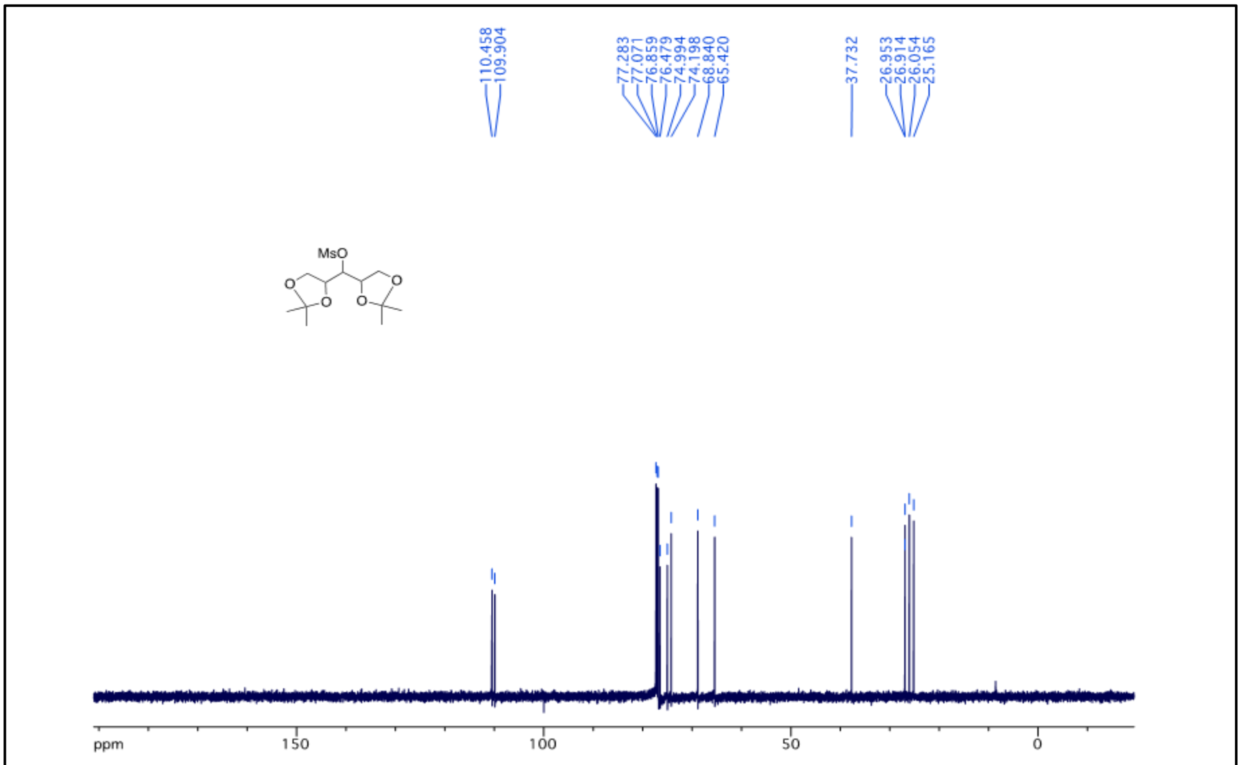

^{13}C NMR of Compound **2**

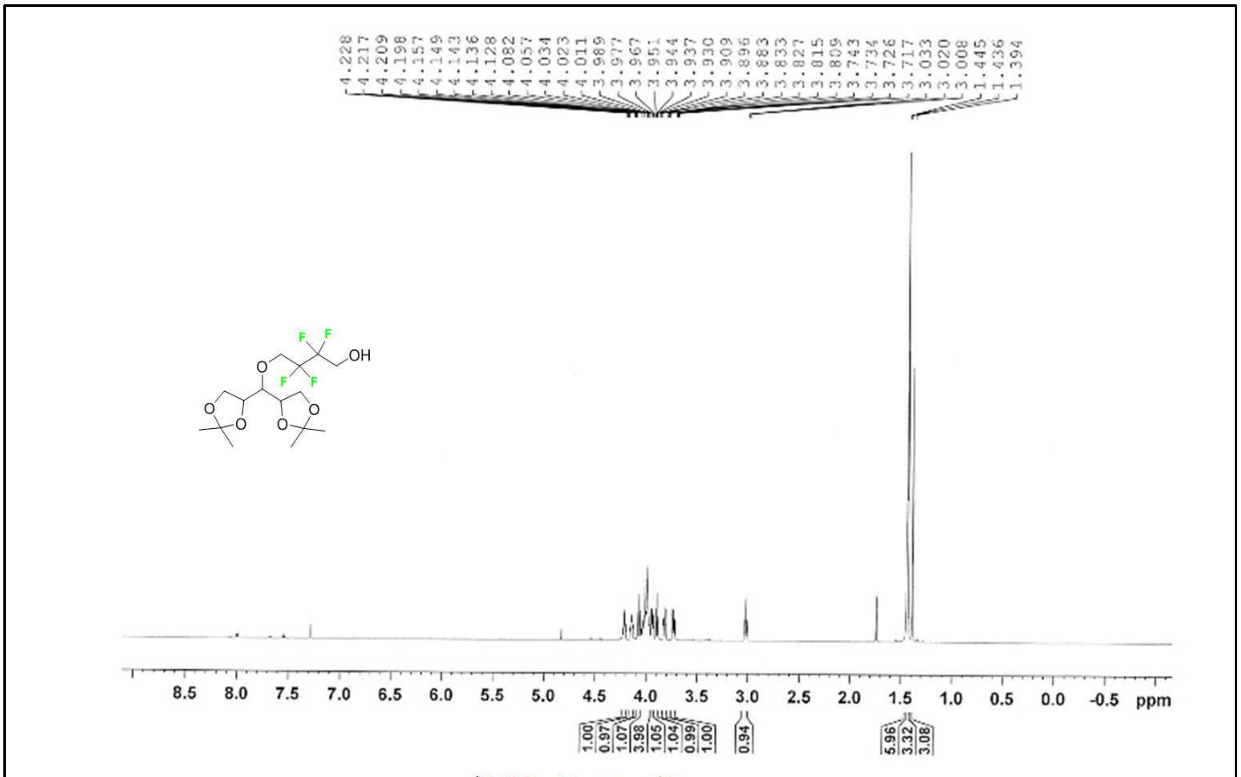

^{1}H NMR of Compound **3**

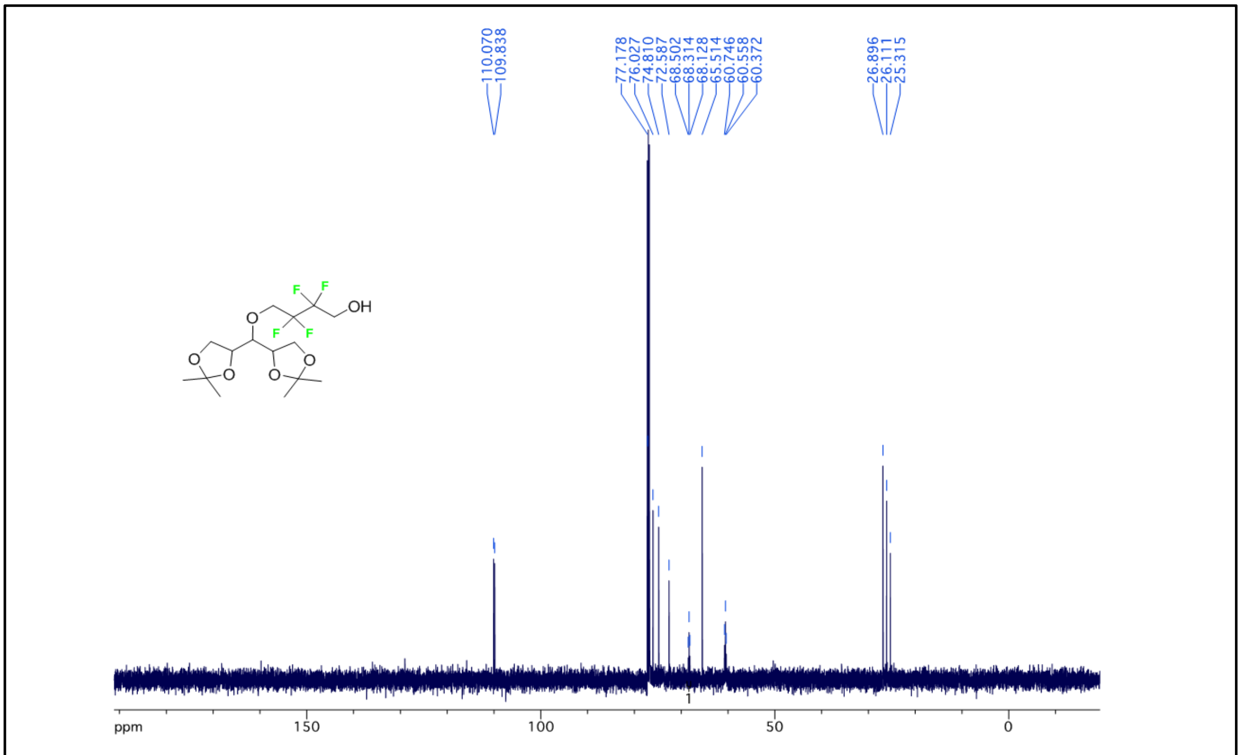

^{13}C NMR of Compound **3**

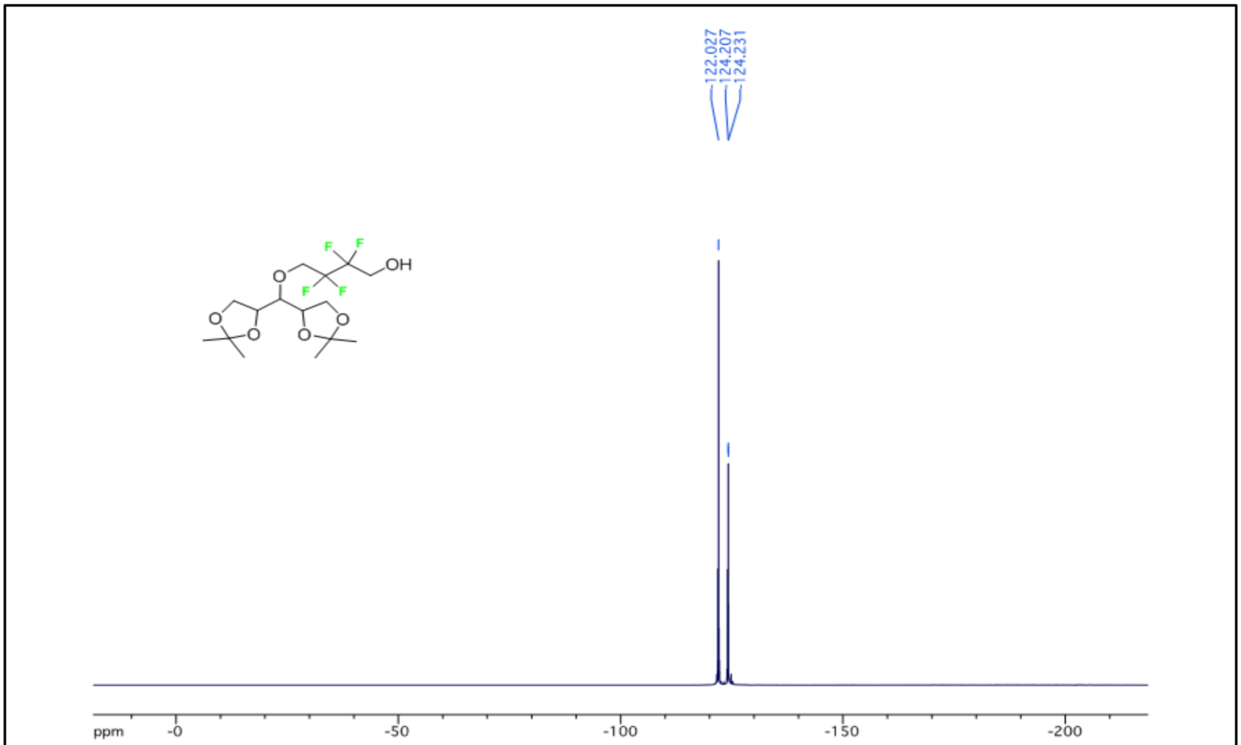

^{19}F NMR of Compound **3**

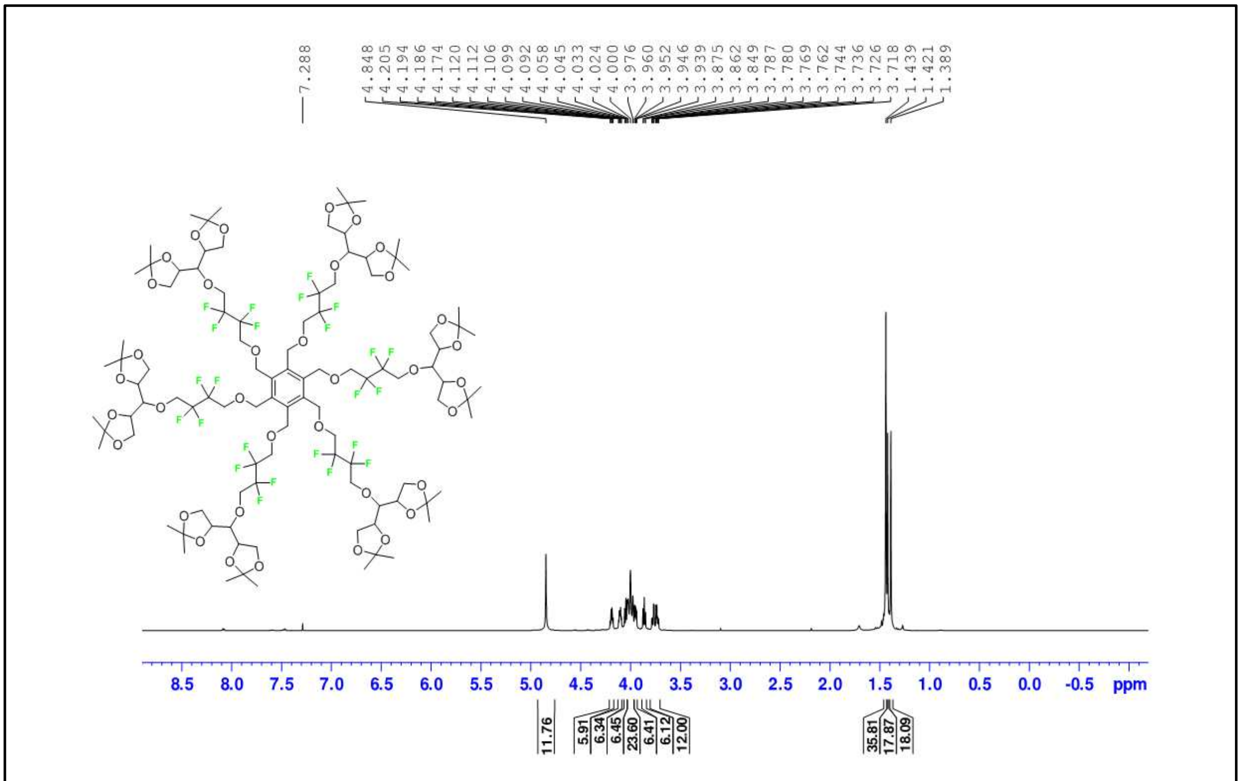

^{13}H NMR of Compound **4**

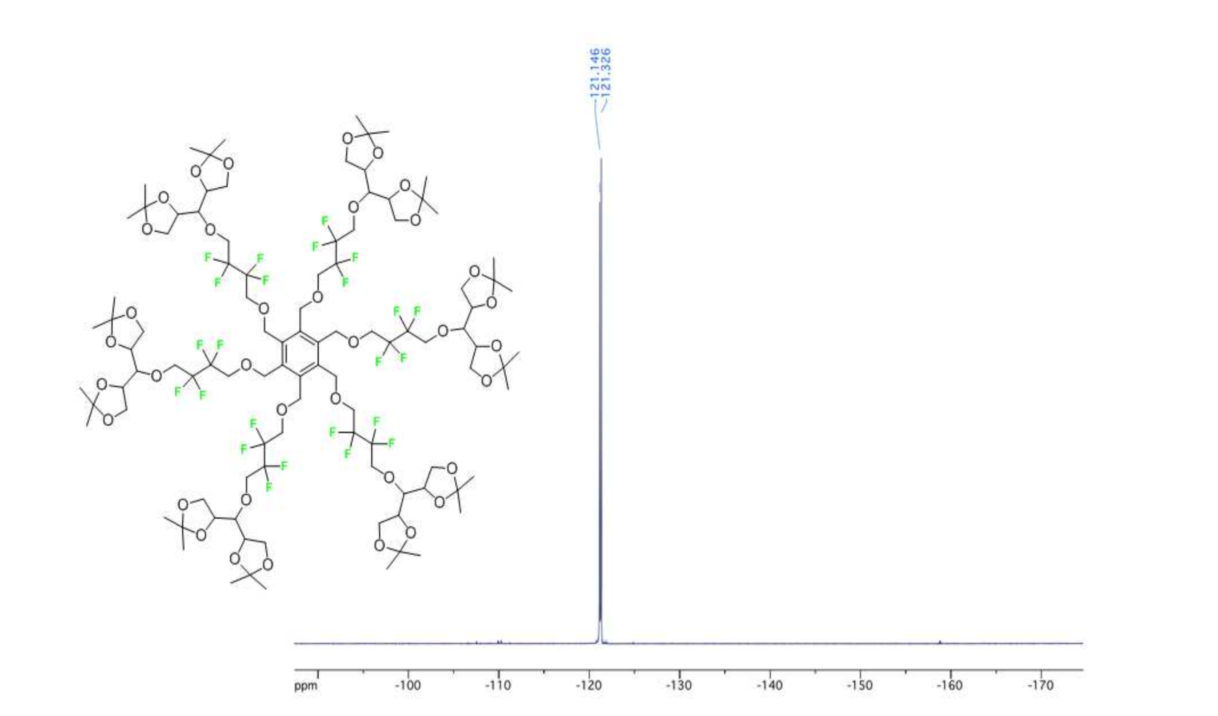

^{19}F NMR of Compound **4**

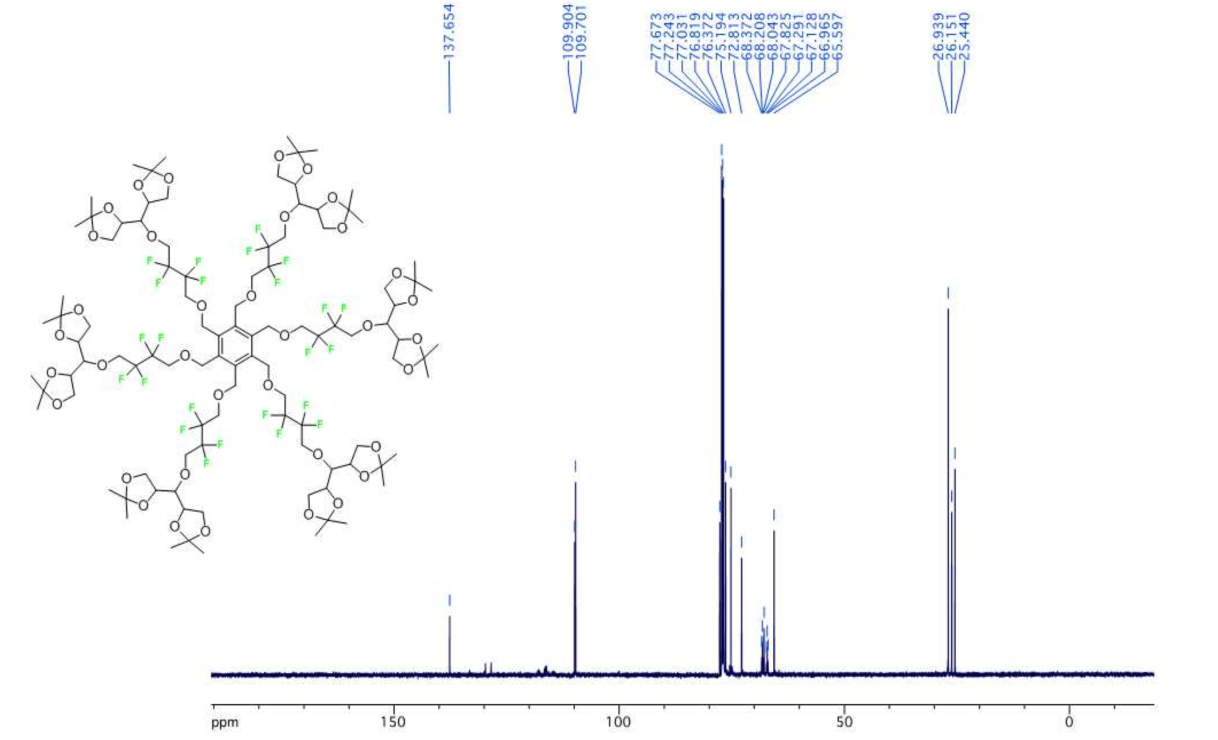

^{13}C NMR of Compound **4**

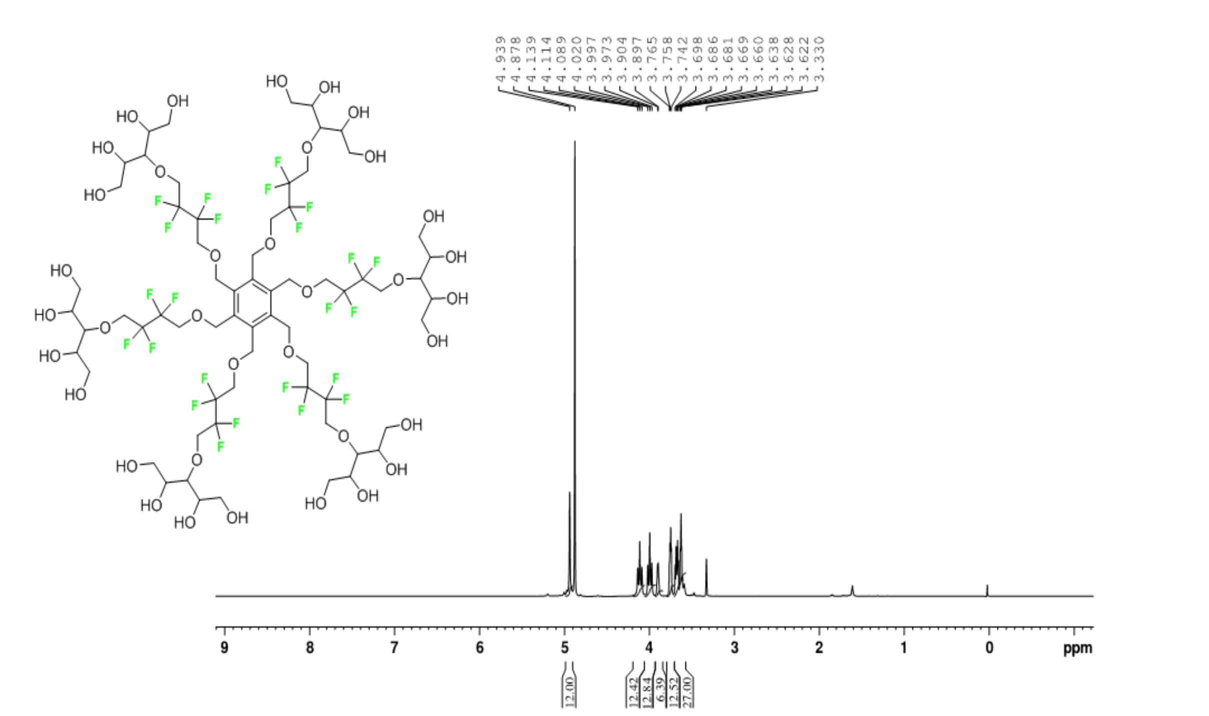

^{1}H NMR of Compound **ET1084**

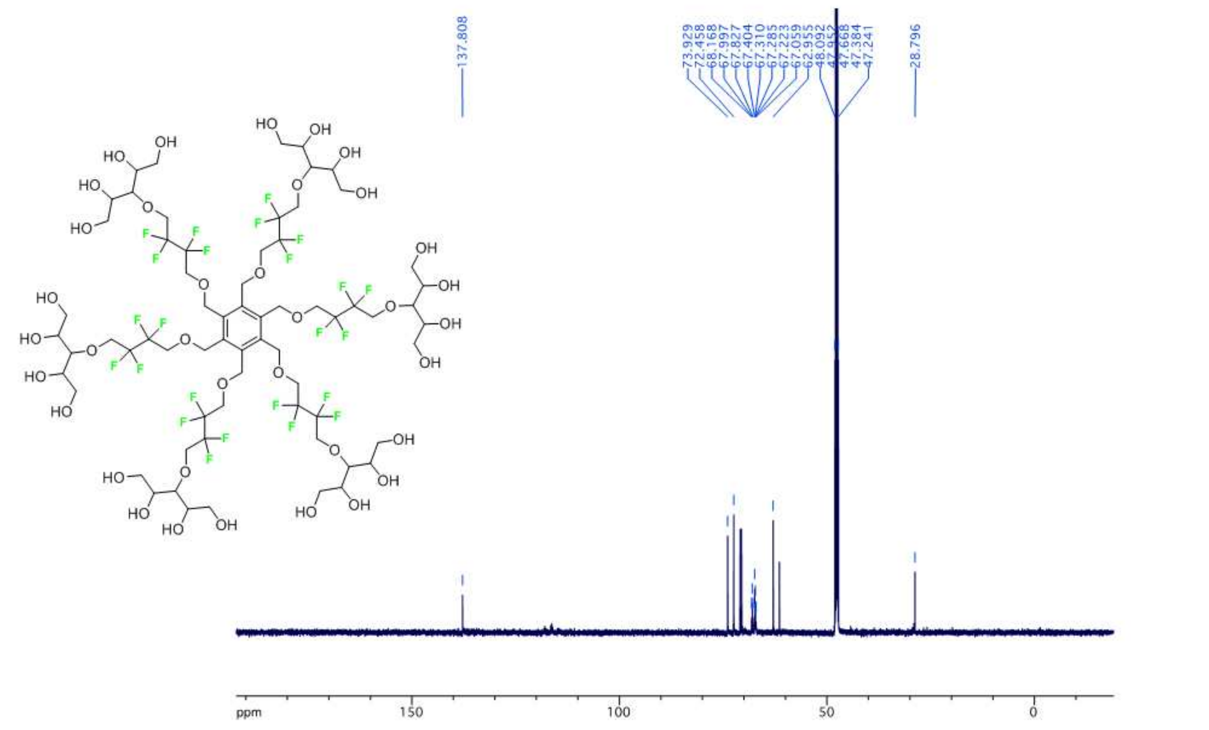

^{13}C NMR of Compound **ET1084**

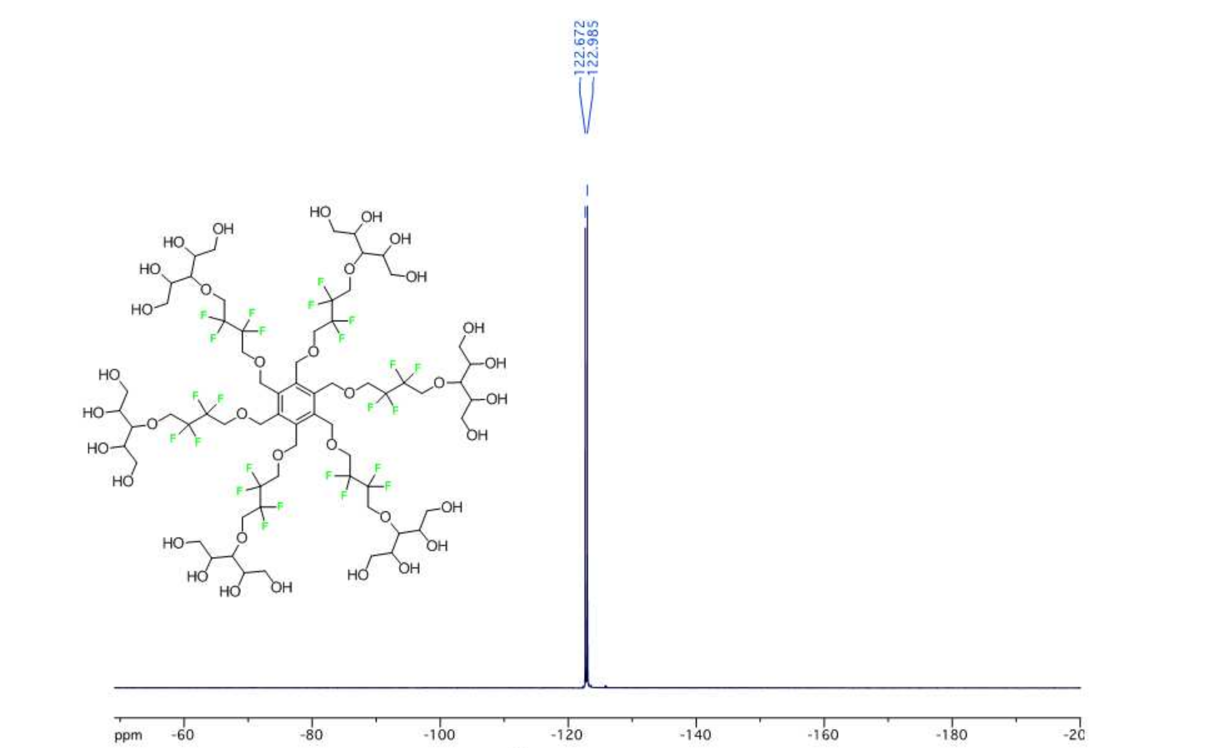

^{19}F NMR of Compound **ET1084**

S2. Determination of optimal concentration range to evaluate the ^{19}F MRI properties of **ET1084**

Preliminary *in vitro* phantom studies were performed on a 1 Tesla (1T) MRI instrument to evaluate the effect of **ET1084** concentration on ^{19}F signal. The parent compound was diluted in deionized water to obtain samples with concentrations of 87.5, 175, 250, 312.5 and 350 mM. The compound dissolved readily to give clear solutions (with minimal warming) at concentrations <250 mM. Upon cooling to room temperature, these solutions remained clear. Solutions with **ET1084** concentration >250 also gave clear solutions while warm but became increasingly turbid upon cooling to room temperature with increasing concentration. Dynamic Light Scattering (DLS) measurements of the solutions did not show a measurable autocorrelation function at concentrations below 250mM. At higher concentrations, as saturation occurs, large undissolved particles exist. This suggested a saturation point of about 250 mM. Imaging was performed on a 1T (permanent magnet) MRI scanner (M2 system, Aspect Imaging, Shoham, Israel), incorporating a 35 mm dual channel (^{1}H and ^{19}F) transmit-receive RF volume coil). ^{19}F MRI scans were acquired using a spin-echo sequence with the following scan parameters: echo time

(TE) = 20 ms, repetition time (TR) = 800 ms, flip angle = 90◦, chemical shift center = -121 ppm, chemical shift selection bandwidth = 20 ppm, slice thickness = 2 mm, field of view = 48 mm, number of slices = 3, matrix = 48 x 48, dwell time = 40 µs, acquisition plane = coronal; in-plane resolution = 1 x1 mm^2, scan time ~ 4 minutes. Number of signal averages (NSA) was varied to understand its effect on image quality. Dicoms obtained from scans were processed using the OsiriX v.5.8.5 software (Pixmeo SARL, Bernex, Switzerland) and signal-to-noise ratio (SNR) for each concentration was calculated from the mean image pixel value as follows:

$$SNR = \frac{\text{Signal(Fluorine)}}{\text{SD(Background)}}$$

Figure **S2.1A** shows representative obtained from ^{1}H and ^{19}F scans with different signal averages. A plot of SNR against concentration of the molecule (Figure **S2.1B**) shows increasing signal with concentration of the molecule to a maximum at about 250 mM and a drop off thereafter. Similarly, Phantoms with concentrations of the molecule at 1, 5, 10, 25, 50, 100, 200 and 250 mM were tested at 9.4T. A Multi Slice Multi Echo (MSME) MSME scan protocol (Excitation bandwidth = 2000 Hz, TR = 2000 ms, TE = 8.95 ms, scan time = 10 min 40 s). Dicoms obtained from scans were processed using the OsiriX v.5.8.5 software (Pixmeo SARL, Bernex, Switzerland) and SNR obtained as described above. A plot of SNR of the ensuing images against concentration of **ET1084** (Figure **S2.2**) also shows increase of SNR with increasing concentration which appears to a maximum at 200 mM and a drop thereafter. A concentration range between 0 - 200 mM was therefore adopted as the optimal range to characterize **ET1084**.

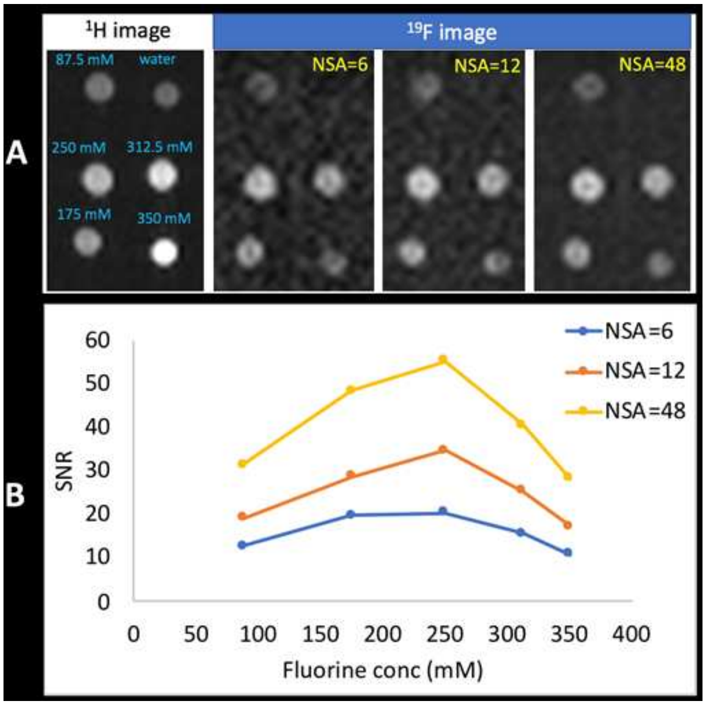

Figure S2.1. Effect of concentration on ^{19}F signal-to-noise ratio (SNR) at 1T. **A**) Plot of SNR versus fluorine concentration for different number of signal averages (NSA). **B**) Representative proton (^{1}H) and fluorine (^{19}F) images demonstrating the effect concentration of molecule and NSA on visibility of fluorine phantoms. Values in proton image indicate concentration **ET1084** (mM).

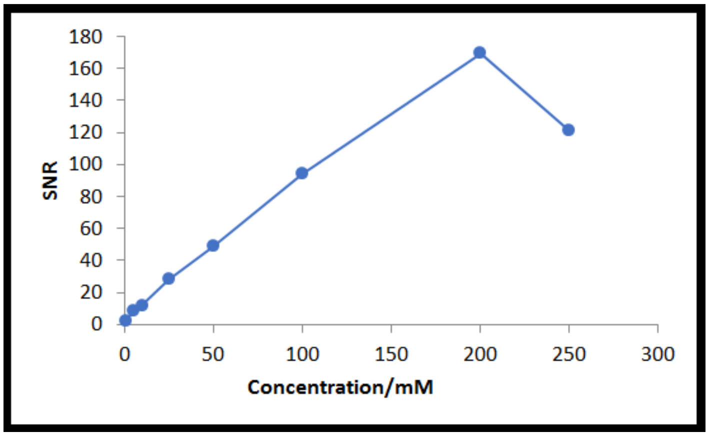

Figure S2.2. SNR of ^{19}F MRI phantoms at 9.4T increase linearly with increasing concentration but drops off at concentrations > 200 mM.
